# Sensing Responses Based on Transfer Characteristics of InAs Nanowire Field-Effect Transistors

**DOI:** 10.3390/s17071640

**Published:** 2017-07-16

**Authors:** Alex C. Tseng, David Lynall, Igor Savelyev, Marina Blumin, Shiliang Wang, Harry E. Ruda

**Affiliations:** 1 Centre for Advanced Nanotechnology, University of Toronto, 170 College Street, Toronto, ON M5S 3E4, Canada; alexc.tseng@mail.utoronto.ca (A.C.T.); david.lynall@mail.utoronto.ca (D.L.); igor.saveliev@utoronto.ca (I.S.); marina.blumin@utoronto.ca (M.B.); 2 Department of Materials Science and Engineering, University of Toronto, 184 College Street, Toronto, ON M5S 3E4, Canada; 3 Defence Research and Development Canada Suffield, Medicine Hat, AB T1A 8K6, Canada; shiliang.wang@drdc-rddc.gc.ca

**Keywords:** nanowire, sensor, field-effect transistor, InAs, adsorption

## Abstract

Nanowire-based field-effect transistors (FETs) have demonstrated considerable promise for a new generation of chemical and biological sensors. Indium arsenide (InAs), by virtue of its high electron mobility and intrinsic surface accumulation layer of electrons, holds properties beneficial for creating high performance sensors that can be used in applications such as point-of-care testing for patients diagnosed with chronic diseases. Here, we propose devices based on a parallel configuration of InAs nanowires and investigate sensor responses from measurements of conductance over time and FET characteristics. The devices were tested in controlled concentrations of vapour containing acetic acid, 2-butanone and methanol. After adsorption of analyte molecules, trends in the transient current and transfer curves are correlated with the nature of the surface interaction. Specifically, we observed proportionality between acetic acid concentration and relative conductance change, off current and surface charge density extracted from subthreshold behaviour. We suggest the origin of the sensing response to acetic acid as a two-part, reversible acid-base and redox reaction between acetic acid, InAs and its native oxide that forms slow, donor-like states at the nanowire surface. We further describe a simple model that is able to distinguish the occurrence of physical versus chemical adsorption by comparing the values of the extracted surface charge density. These studies demonstrate that InAs nanowires can produce a multitude of sensor responses for the purpose of developing next generation, multi-dimensional sensor applications.

## 1. Introduction

The ability to detect the identity and quantity of certain volatile organic compounds like ethanol or acetic acid finds key applications in areas such as process control [1], environmental monitoring [2,3] and healthcare [4]. The latter is particularly interesting as the ability to sense acetic acid is relevant to diagnoses using so-called molecular “biomarkers” in the exhaled breath of patients with cystic fibrosis or gastroesophageal reflux disease [5,6]. However, the established techniques (e.g., mass spectrometry) are lab-scale or the sensor materials require high temperature operation [1,2] and are hence not conducive to real-time or point-of-care testing. To address these challenges, a significant body of work has been produced on biological and chemical sensors using field-effect transistors (FETs) based on semiconducting nanowires (NWs) [7,8,9,10,11,12,13].

Semiconductors are, by virtue of their energy band structure, highly suited for this function. It is well known that the interaction between the surface of a semiconductor and adsorbed chemical species produces a change in the local band structure, whether electrostatically via the charge of adsorbed molecules or directly by altering the distribution of allowed electron states at the surface [14,15]. By utilizing an FET configuration, this interaction can be detected as a change in carrier transport (e.g., via conductance) and behaviour in applied electric fields. To obtain a sensing response, the typical approach is to correlate conductance changes to analyte concentration [11,13]. For NWs in particular, it has been shown that conductance can be greatly modulated by the (de)occupancy of just a single charge in surface trap states [16,17]. Hence, this capability for high sensitivity, along with arguments such as increased surface area-to-volume ratio and uniform gating by surface charges due to comparable sizes of the Thomas–Fermi screening length and NW diameter [17,18], draws the state-of-the-art towards semiconducting nanowire materials.

A majority of work in this field has focused on biological applications using silicon [19,20,21,22], partly due to the ability to functionalize its native oxide surface for label-free biological sensing; while for industrial applications, metal-oxide (i.e., II-VI semiconductor) gas sensors are widely favoured for operability in high temperatures [23,24]. However, this trend leaves higher performance III-V semiconductors relatively unexplored. Indium arsenide (InAs) is notable in particular due to high values of electron mobility [25] and an intrinsic surface accumulation of electrons due to the presence of a large density of surface states [26,27]. The former lends itself to producing sensors that operate well at ambient temperatures, which is vital to biological applications. The latter plays a key role in the fabrication of InAs NWFETs, allowing for facile formation of ohmic contacts [28] to produce devices with high signal-to-noise ratios. To date, devices based on InAs have shown responses to alcoholic vapours, H_2_O, NO and NO_2_ [29,30,31,32]. Using an ion-sensitive (IS) FET structure, pH and proteins have also been detected in solution [33].

While most reports of NWFET sensors attribute the sensing response to a conductance change caused by alterations of surface potential (i.e., a gating effect due to adsorption of charged analytes) [18,21,33,34], few independent groups have discussed the impact of the sensing interaction on other transport parameters obtained by field-effect measurements (e.g., carrier mobility, threshold voltage, subthreshold swing) [29,32,35,36,37]. This is perhaps due to the prevalence of biosensing work that employs dielectric-capped ISFETs as compared to gas/chemical sensing that typically uses FETs with exposed channels and reactive surface states. The latter is the relevant case for as-grown InAs NWs, due to the non-stoichiometry of its native oxide [38]. However, inconsistencies in the literature [31,32] point to the need for studies of the sensing mechanisms in InAs NWFETs.

Here, we report on transient current and field-effect measurements for an InAs multi-NWFET under inert N_2_ and vaporized chemical environments. In particular, the response of our device to vapours of acetic acid demonstrates the ability of a reversible chemical reaction at the surface to modulate electronic transport and produce a sensing response in select transport parameters.

## 2. Materials and Methods

**Figure 1:**
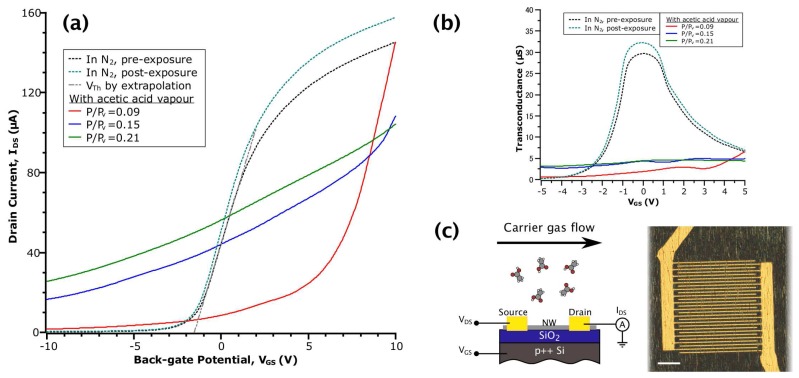
(**a**) $I_{\mathrm{DS}}$-$V_{\mathrm{GS}}$ curves of InAs nanowire FET (NWFET) in various environments. The grey dashed line shows the method of obtaining $V_{\mathrm{Th}}$ by extrapolation of a linear fit; (**b**) Transconductance versus $V_{\mathrm{GS}}$ obtained by numerical differentiation of the data in (a); (**c**) Schematic diagram of the NWFET structure (left) and optical micrograph of the device (right) with a scale bar of 20 µm.

InAs NWs were grown using molecular beam epitaxy through the vapour-liquid solid mechanism on GaAs (100) substrates seeded with gold droplets [39]. These NWs had a wurtzite crystal structure and an average diameter of (31 ± 7) nm. Subsequently, NWs were aligned and transferred onto degenerately doped p-type Si substrates with 100 nm-thick oxide via a mechanical contact printing process similar to [40]. Inter-digitated electrical contacts were then defined by electron beam lithography on a spin-coated PMMA resist layer. Ti (20 nm)/Au (90 nm) metal was deposited onto the substrate following an evaporation and lift-off procedure described elsewhere [41]. The inter-digital spacing defines the channel length and was nominally 2 µm; however, following metal deposition, the average length was measured as 1.8 µm. Figure 1c shows an optical micrograph of a device fabricated in this manner containing approximately 1600 NW channels in parallel.

Device substrates were bonded to ceramic chip carriers and placed inside a purpose-built environmental chamber, which was open to atmosphere at its exhaust. Analytes of various chemical species were passed into the chamber in the vapour phase by bubbling N_2_ carrier gas through a bubbler containing the analyte in liquid form. A water bath was used to maintain the bubbler contents at the ambient temperature (~295 K) during vapourization. The vapour stream was then diluted by mixing with a larger flow of the carrier gas prior to admittance to the chamber. Both streams were manually controlled using correlated flow-tube rotameters. A nominal total flow rate to the chamber was set at ~1.4 SLPM (standard litres per minute), and each stream was adjusted to produce the desired concentration (in terms of $P/P_{v}$: the ratio of partial pressure in the input stream to equilibrium vapour pressure) of vaporized chemical species. The following chemicals were used as supplied: glacial acetic acid (Caledon Laboratories Ltd., Georgetown, ON, Canada; reagent grade ≥ 99.7%), 2-butanone (Sigma-Aldrich, St. Louis, MO, USA; ACS reagent grade ≥ 99.0%) and methanol (Caledon Laboratories Ltd.; HPLC grade ≥ 99.8%).

Electrical measurements were performed using an HP4140B picoammeter and customized LABVIEW controls, which simultaneously-measured source-drain current, $I_{\mathrm{DS}}$, while providing a constant DC source-drain bias ($V_{\mathrm{DS}}$ = 10 mV) and varied back-gate potential, $V_{\mathrm{GS}}$. FET transfer characteristics ($I_{\mathrm{DS}}$-$V_{\mathrm{GS}}$ curves) were obtained by sweeping the back-gate from positive (accumulating) to negative (depleting) potentials at a rate of 86 mV/s. To account for drift in the FET behaviour due to device history, transfer curves were obtained prior to exposure to analyte as a baseline for comparison. Notably, transport measurements on the same device over the course of the study showed a return to consistent values given sufficient time spent in inert N_2_ atmosphere. Transient current measurements were taken at 1 s intervals, with the device unbiased by the back-gate. Despite earlier suggestions of enhanced sensitivity when biased to a threshold [42], we found the unbiased, equilibrium condition to best facilitate analysis of transient current data, as this avoided the sensor drift caused by the influence of a varying nanowire capacitance due to adsorption (see Supporting Information, Figure S1).

For the purpose of extracting the apparent field-effect mobility, $\mu_{\mathrm{FE}}$, data from the linear region of the respective $I_{\mathrm{DS}}$-$V_{\mathrm{GS}}$ curves were used. As the curves tend to inflect through this region, this corresponds with the peak values of transconductance ($\partial I_{\mathrm{DS}}/\partial V_{\mathrm{GS}}$) as determined by numerical differentiation. The following equations were employed [41]:

(1) $$\begin{array}{c} \mu_{\mathrm{FE}}=\left(\frac{\partial I_{\mathrm{DS}}}{\partial V_{\mathrm{GS}}}\right)\frac{L^{2}}{V_{\mathrm{DS}}C_{\mathrm{gate}}} \end{array}$$

(2) $$\begin{array}{c} C_{\mathrm{gate}}=\frac{2\pi \varepsilon_{0}\varepsilon_{\mathrm{eff}}}{\mathrm{arccosh}(\left(t_{\mathrm{ox}}+R\right)/R)} \end{array}$$

where *L* is channel length, *R* is NW radius, $t_{\mathrm{ox}}$ is back-gate dielectric thickness and $\varepsilon_{0}\varepsilon_{\mathrm{eff}}$ is the effective permittivity of the SiO_2_ gate dielectric modified by the geometric arrangement [41]. Gate capacitances were calculated in (2) by considering a parallel configuration of each NW channel formed and applying a Gaussian distribution to the NW radii. The values of $\mu_{\mathrm{FE}}$ determined in this manner serve as an approximation of the intrinsic carrier mobility, μ. Threshold voltages, $V_{\mathrm{Th}}$, were determined by extrapolation of the linear region to the abscissa (see Figure 1a). For some of the data, it is not clear that the classical model of MOSFET transfer behaviour is applicable; hence, the above values are rather determined in a low-field condition ($V_{\mathrm{GS}}∼0V$) or otherwise omitted.

## 3. Results and Discussion

### 3.1. FET Transfer Curves in Acetic Acid

Figure 1a shows the transfer curves from the same device collected at different stages of an acetic acid sensing experiment. Prior to taking each measurement, the transient current response was given time to reach an effective equilibrium (i.e., unchanging on a scale of 10 min). Referring to the curves in inert N_2_ gas (dotted lines), both before and after exposure to a vapour of acetic acid, we observe the field-dependent behaviour typical of an n-type NWFET: reduced transconductance (or mobility) in highly accumulating and depleting fields and approximately linear behaviour in the low-field regime [25], which results in a sigmoidal appearance. After returning to N_2_ post-exposure, the device had the same $V_{\mathrm{Th}}$ of −1.6 V, with a slight increase in $I_{\mathrm{DS}}$ (equivalently, conductance) due to increased $\mu_{\mathrm{FE}}$ (indicated by an 8% increase in peak transconductance between the N_2_ curves in Figure 1b). Together, these observations suggested a reduction in the degree of carrier scattering while maintaining the same charge neutrality level. This is perhaps due to a reduction of surface roughness or the removal of an equal amount of positively and negatively-charged scattering centres caused by the interaction of acetic acid and the NW native oxide. These transfer curves in N_2_ contrast with data collected in the presence of acetic acid vapour, whereby it is apparent that the adsorption of acetic acid on the NW surface drastically changes the NWFET field-dependent behaviour.

Considering the data in the three relative concentrations of acetic acid (the red, blue and green solid line curves in Figure 1a), the NWFET exhibited a loss of depletion behaviour. That is, the gate potential became much less effective at reducing electron density in the NW channel. This is reflected in the values of off current (defined here as $I_{\mathrm{off}}$ = $I_{\mathrm{DS}}$(−10 V)), which are increased by one to two orders of magnitude over the values in N_2_, depending on vapour concentration. Additionally, values of transconductance in the low-field regime are decreased by seven- to 15-fold (see Figure 1b). We attribute these behaviours to the creation of donor-like states at the NW surface due to the interaction with acetic acid. This stands in contrast to the acceptor-like states proposed for NO_2_ adsorption [31]. Typically, as $V_{\mathrm{GS}}$ is swept, the Fermi level ($E_{F}$) in the NW channel is moved from the conduction band (CB) into the band gap, according to the change in electron density. However, if a population of donor-like states exists near the CB, when $E_{F}$ meets the level of these states during the sweep, electrons will be transferred into the channel. Because the electron density is now influenced extrinsically, this effectively reduces the rate of change of $E_{F}$ with respect to the gating potential (i.e., $E_{F}$ is pinned) and results in lowered transconductance. At the same time, the donated electrons contribute to the observed increase in off current.

The curve at acetic acid $P/P_{v}$ = 0.09 has a different shape than the other acetic acid curves: it starts at the same value of $I_{\mathrm{DS}}$ as in the pre-exposure N_2_ curve, but quickly drops as the sweep continues. To explain this behaviour, we consider the equilibrium of the adsorption reaction that produces the donor-like states. In accumulating fields, $E_{F}$ (alternatively, the chemical potential of electrons) is raised above the level of the states. This shifts the adsorption reaction in the reverse direction (i.e., desorption) and results in transport behaviour that at first resembles a bare NW. Fan et al., described a similar process, but with the acceptor-like states formed by adsorption of NO_2_ on ZnO NW sensors in depleting fields [43]. As the sweep continues, $E_{F}$ is lowered, and adsorption again dominates. With increasing vapour concentration, this effect is minimized as the rate of desorption is countered by the increase in the total number of adsorbed molecules. Together, the field-dependent behaviour of our InAs NWFET in acetic acid point towards the creation of donor-like states near the CB as the principal mechanism that produces measurable changes that can be utilized for sensing.

### 3.2. Physical versus Chemical Adsorption

**Figure 2:**
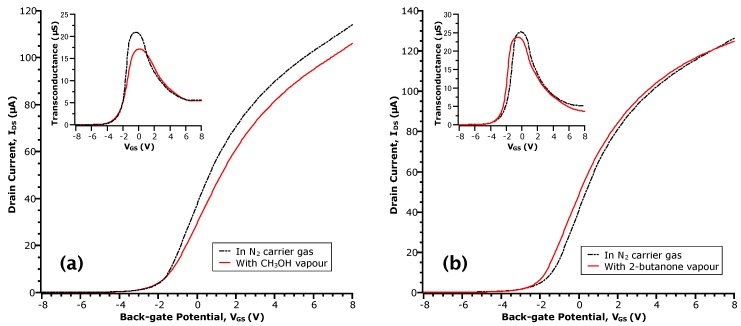
$I_{\mathrm{DS}}$-$V_{\mathrm{GS}}$ curves of InAs MWFET in: vapours of (**a**) methanol (CH_3_OH) at a concentration of $P/P_{v}$ = 0.27; and (**b**) 2-butanone at a concentration of $P/P_{v}$ = 0.16. The transfer curve taken in N_2_ prior to each exposure is shown in the dotted-dashed line. Insets are the respective transconductance curves obtained by numerical differentiation of the data.

The most probable source of the aforementioned donor-like states is the carboxylic acid moiety of the acetic acid molecule, as compared to the aliphatic nature of the remaining methyl group. To test this hypothesis, we collected transfer curves of this device in the presence of methanol (CH_3_OH), which contains a hydroxyl moiety, and 2-butanone, which contains a carbonyl moiety. In this manner, we aimed to test the effect of the sub-parts of the carboxylic acid moiety in comparison to the whole. 2-butanone was chosen for its lower vapour pressure relative to a simpler ketone, acetone, which evaporated too rapidly for practical purposes. Figure 2 gives the transfer curves at $P/P_{v}$ = 0.27 for methanol and $P/P_{v}$ = 0.16 for 2-butanone. Immediately, we can see the lack of drastic change as with the transfer curves in acetic acid.

For the case of methanol (Figure 2a), no shift of the threshold voltage ($V_{\mathrm{Th}}$ = −1.8 V) relative to N_2_ is observed. Moreover, there is a ~17% decrease in $\mu_{\mathrm{FE}}$ (see the peak transconductance in the inset of Figure 2a), perhaps due to increased carrier scattering. At a given $V_{\mathrm{GS}}$, this results in a reduction of $I_{\mathrm{DS}}$ in the methanol transfer curve relative to N_2_. These results appear in contrast to the work by Du et al. for other alcoholic vapours on InAs NW surfaces [29]. However, the sensor response of NWFETs is keenly dependent on the distribution of surface states that interact with adsorbed molecules. This is, in turn, contingent on differences in the materials and processing steps and is reflected in the transfer curves of our devices versus theirs. In the 2-butanone case (Figure 2b), we observe a shift of $V_{\mathrm{Th}}$ by −0.5 V (i.e., towards negative $V_{\mathrm{GS}}$). However, unlike methanol, the value of $\mu_{\mathrm{FE}}$ remains relatively unchanged such that the transfer curve in 2-butanone appears to shift altogether. Moreover, neither methanol or 2-butanone showed a significant influence on the values of $I_{\mathrm{off}}$, suggesting the lack of electron transfer to the NW channel and a physical interaction of these analytes with the surface rather than a chemical one.

An extended qualitative description comparing the transfer curves in acetic acid versus methanol and 2-butanone vapour would require an account of the nature of adsorbate bond formation, carrier scattering, surface dipole moments and steric effects, to name a few. To fully address these topics exceeds the scope of the present work. However, from the data, we can extract a simple quantitative measure to distinguish between physical and chemical interactions in the form of an areal surface charge density, $Q_{S}$. In the Volkenstein model of adsorption and catalysis on semiconductor materials, the distinction between physical and chemical adsorption is given in terms of the formation of neutral or charged adsorptive electron states, respectively [15]. Hence, the differential of $Q_{S}$ before and during analyte exposure ($\Delta Q_{S}$) can be related to changes in surface charge due to adsorption. To extract $Q_{S}$, we apply the following model, similar to that used to determine MOSFET subthreshold swing, $S_{S}$:

(3) $$\begin{array}{c} S_{S}=\ln\left(10\right)\left(\frac{kT}{e}\right)\left(1+\frac{C_{\mathrm{NW}}}{C_{\mathrm{gate}}}\right) \end{array}$$

(4) $$\begin{array}{c} Q_{S}=\frac{C_{\mathrm{NW}}\left(V_{\mathrm{GS}}-V_{\mathrm{Th}}\right)}{e2\pi RL} \end{array}$$

where kT/e is the thermal voltage and CNW is the capacitance due to the distribution of charge across the NW. The denominator of (4) is the total active surface area of the NWFET, assuming a cylindrical NW. $Q_{S}$ is given in units of *e* ${\mathrm{cm}}^{-2}$. In (4), $V_{\mathrm{GS}}$ is assumed to be zero at the charge neutrality level; hence, $\left(V_{\mathrm{GS}}-V_{\mathrm{Th}}\right)$ represents the potential required to bring enough mobile charges into the NW channel to balance the surface charge. Values of $S_{S}$ can be determined graphically from semi-log plots of $I_{\mathrm{DS}}$-$V_{\mathrm{GS}}$ (Supporting Information, Figure S2) and used to find values of $C_{\mathrm{NW}}$.

In (3) and (4), it is clear that $S_{S}$ and $Q_{S}$ are directly proportional to $C_{\mathrm{NW}}$. Subsequently, a change in surface charge due to adsorption can be modelled as an additional parallel capacitance (i.e., $C_{\mathrm{NW}}=C_{\mathrm{bulk}}+C_{\mathrm{ads}}$), similar to an interface state capacitance [25]. Thus, we expect the values of $S_{S}$ and $Q_{S}$ to increase if chemical adsorption is dominant. For our NWFET in N_2_ (i.e., without adsorbates), we find $Q_{S}\approx 1.6\times 10^{13}e{\mathrm{cm}}^{-2}$. Given that in this case, each *e* of charge corresponds directly to a single allowed electron state, this value is in good agreement with previous work on the surface state density of InAs surfaces [27,41]. The values of $Q_{S}$ in various analytes are summarized in Table 1 along with other transport parameters.

For methanol and 2-butanone, the values of $\Delta Q_{S}$ are on the order of 2 to 3 × $10^{12}e{\mathrm{cm}}^{-2}$, that is, only about 10% of $Q_{S}$ in N_2_. Considering that their equilibrium vapour pressures are an order of magnitude greater than that of acetic acid, these results are in spite of having greater coverage at similar $P/P_{v}$. For acetic acid, $\Delta Q_{S}$ is on the order of 1 to 2 × $10^{14}e{\mathrm{cm}}^{-2}$, a 10-fold increase compared to N_2_. This order of magnitude change in $Q_{S}$ implies that adsorbate charge will have a dominating effect on the transport behaviour of the NWFET in acetic acid, which agrees with our observations of the transfer curves. Furthermore, we can expect the magnitude of $\Delta Q_{S}$ to increase with vapour concentration, as more molecules are available to chemically adsorb to the surface.

From these extracted values of $\Delta Q_{S}$, we can see two orders of magnitude change ($10^{12}\;∼\;10^{14}$) between methanol and 2-butanone versus acetic acid. By this, we have a clear distinction between a physical and chemical adsorption. In terms of field-dependent behaviour, we observe that a physical interaction largely preserves the features of the transfer curve, as is the case with methanol and 2-butanone. By comparison, this strongly suggests that the observed behaviour of the InAs NWFET in acetic acid stems from a chemical reaction (i.e., chemisorption) with the NW surface.

### 3.3. Current Transients in Acetic Acid

**Figure 3:**
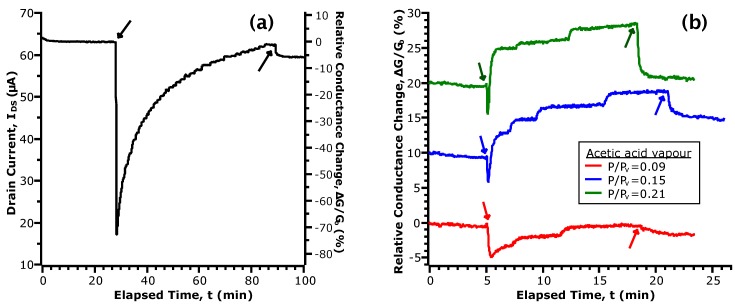
Selected portions of the transient response of $I_{\mathrm{DS}}$ upon exposure to acetic acid vapour. The downward and upward facing arrows indicate the time that vapour was introduced and removed from the chamber, respectively. (**a**) shows the initial response of our device; (**b**) shows the response in three concentrations of vapour, normalized by the baseline conductance prior to exposure. For clarity, the curves are shown with an offset of 10% relative to each other.

We now turn to time-domain measurements of the FET current in an atmosphere of acetic acid (shown in Figure 3) to gain insight into the chemisorptive interaction. This experiment was designed to expose vapour in an order of alternating high and low concentrations to distinguish against the possibility of the accumulation of analyte giving rise to the observed effects. To facilitate the discussion, the current-time trace has been cropped to show regions of interest. The continuous data can be found in the Supporting Information, Figure S3. We note that the current fluctuations observed in the data stem from manual control of the rotameters as they are adjusted to compensate for drift in gas flow in order to maintain the concentration set-point. Though this is an undesirable aspect of our experimental setup, it also demonstrates the sensitivity of our device to small changes in vapour pressure.

Figure 3a shows the current transient upon initial exposure to acetic acid vapour. Here, we note two sequential phenomena: first, a large, sharp decrease in $I_{\mathrm{DS}}$; followed secondly by a gradual restoration of current to the initial values. While analysis of the transfer curves showed the formation of donor-like states via chemisorption of acetic acid, the transient behaviour points towards this as being a two-step process. To account for the initial drop in $I_{\mathrm{DS}}$, we propose that the first step is an acid-base reaction between acetic acid and the amphoteric native oxides of InAs. The formation of acetate anions ([CH_3_COO]−) by dissociation of the hydrogen from the hydroxyl group results in a population of negatively-charged species on the NW channel. These act as repulsive scattering centres to greatly decrease conductance [44]. Thereafter, the gradual increase of current is given by the formation of donor-like states at the NW surface with a time dependence for electron transfer. In this second step, we further propose that the initial acid-base reaction is followed by a redox reaction between acetate and the native oxide layer. Since arsenic oxides are typically non-stoichiometric and contain both As^III^ and As^V^, with the +5 oxidation state being less stable, reduction is possible by electron transfer from acetate. The rate of these redox reactions contributes to the time dependence of the current transient. Upon removal of acetic acid vapour from the chamber, $I_{\mathrm{DS}}$ responds by quickly returning to a baseline level. We infer from this that the redox reactions are reversible, giving rise to a dynamic equilibrium that is dependent on the partial pressure of acetic acid above the NW surface.

In Figure 3b, we compare the transient current response in different concentrations of acetic acid vapour. These data have been selected due to uniformity of the time spent in N_2_ and vapour. To facilitate comparison, the data have been normalized by the baseline values taken at the start of exposure and are given in terms of a relative change of conductance (i.e., $G=I_{\mathrm{DS}}/V_{\mathrm{DS}}$). First, while we observe the initial drop in conductance as in Figure 3a, the magnitude of the change is greatly decreased (5% vs. 75%). Considering that the reactant in the acid-base reaction is a non-hydrated oxide, it follows that the extent of acetate formation will be limited with repeated exposures. For a greater intervening length of time spent in N_2_, a larger number of active surface sites will be replenished through the desorption of hydrogen. This agrees with our observations of the current transient in the initial exposure and when a longer time was taken between consecutive exposures (see Figure S2). In the comparison between curves in different $P/P_{v}$, we observe two concentration dependent parameters: the time constant of the increasing current and the magnitude of the conductance change after turning off vapour flow to the chamber. While both are related to $P/P_{v}$ by the extent of the proposed redox reaction, the rate of increase of $I_{\mathrm{DS}}$ is strongly affected by the previous state of the device as discussed earlier. Hence, we will now examine the conductance change after turning off vapour flow as it refers to a change between two equilibrium points: with and without the presence of acetic acid vapour.

### 3.4. Correlation of Sensor Response

From our analysis in previous sections, we have identified the relation of some device parameters to the type and extent of analyte interaction with the NW surface (see Table 1). To utilize the InAs NWFET as a sensing device, a clear trend between these parameters and analyte concentration is required. While we observed that values of apparent field-effect mobility, $\mu_{\mathrm{FE}}$, were sensitive to the influence of adsorption on carrier scattering, in the case of acetic acid, we did not find a clear trend in concentration. Likewise, values of the threshold voltage, $V_{\mathrm{Th}}$, could only be extracted when the field-dependent behaviour was not drastically modified by the analyte interaction. Of the remaining parameters, values of off current ($I_{\mathrm{off}}$) from the transfer curves, the change in surface charge density ($\Delta Q_{S}$) extracted from a model of subthreshold behaviour and the relative magnitude of conductance changes ($\Delta G/G_{o}$) in the the transient data showed a trend with acetic acid concentration.

**Figure 4:**
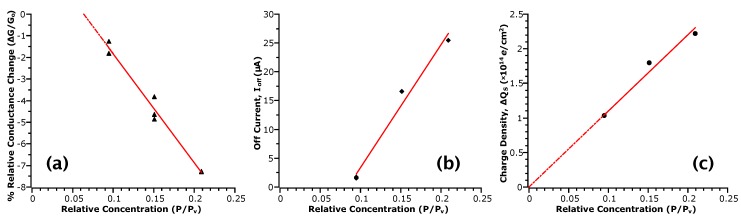
Sensor responses correlated with $P/P_{v}$. Red lines are a linear fit to the data and serve as a guide to the eye. (**a**) is extracted from the relative conductance change before and after removal of analyte from the sensing chamber, where $G_{o}$ is the conductance before; (**b**) is the off current defined as $I_{\mathrm{DS}}$(−10 V) from the $I_{\mathrm{DS}}$-$V_{\mathrm{GS}}$ curves; (**c**) is the differential surface charge density extracted from values of subthreshold swing obtained from the $\log(I_{\mathrm{DS}})$-$V_{\mathrm{GS}}$ curves.

Figure 4 plots the values of $\Delta G/G_{o}$, $I_{\mathrm{off}}$ and $\Delta Q_{S}$ with respect to $P/P_{v}$. From these plots, we indeed observe proportionality between the measurements and analyte concentration, which is linear, suggesting that these values may be used to quantify the sensing response. Considering the sensitivity of the response as the relative change between maximum and minimum points, for an approximately two-fold change in $P/P_{v}$, we see about a six-fold change in $\Delta G/G_{o}$, 14-fold in $I_{\mathrm{off}}$ and two-fold in $\Delta Q_{S}$. Previously, only conductance measures have typically been employed, partly due to the benefit of real-time measurement. However, such a response is not selective of the type of analyte interaction with the NW surface as multiple factors can affect conductance; for example, change in carrier concentration due to gating, change in mobility due to scattering, etc. However, as discussed, other transport parameters can respond to specific phenomena. For instance, $I_{\mathrm{off}}$ responds if the analyte interaction involves a transfer of charge to the channel via adsorption, and large changes in $\Delta Q_{S}$ are indicative of chemical rather than physical adsorption. These results indicate that values of $I_{\mathrm{off}}$ may be twice as sensitive to concentration than the $\Delta G/G_{o}$ response in the range of concentrations tested. In contrast, the $\Delta Q_{S}$ response shows almost a direct one-to-one relationship to $P/P_{v}$. While this measure may appear to be less sensitive, this linearity is likely to extend beyond the lower limit of concentrations tested here. In this case, the limit of detection will depend on the ability to accurately determine the capacitances used in the model.

However, the present study is limited in both the concentration range and complexity of the analytes tested. In real-word applications, such as the detection of breath biomarkers in patients, the analyte is a mixture of species, and competing adsorptive events come into play. Here, especially, having a multitude of sensor responses that are selective of different phenomena is key to obtaining a better understanding of the unknown. The result is a multi-dimensional dataset, which can be interpreted using the aid of machine-learning and feature-reduction techniques [4,45,46]. The established approach to obtain independent sensor responses is to either functionalize the active surface with selective molecules or otherwise change the composition of the material used as the sensor. However, in this study, we have shown that the parameters used as the sensor response can themselves be selective of the phenomena that produce them.

## 4. Conclusions

Through sensing experiments performed with glacial acetic acid, methanol and 2-butanone, we demonstrated the capability of InAs multi-NWFETs to distinguish between adsorptive events of both a physical and a chemical nature. Specifically, we found that differential values of surface charge density ($\Delta Q_{S}$) as determined by the subthreshold behaviour of InAs NWFETs before and during exposure to the analyte were able to distinguish between physical and chemical adsorption. With regards to the chemisorption of acetic acid on the InAs native oxide surface, we proposed a two-part, reversible acid-base and redox reaction that results in the creation of slow donor-like states on the surface. This produced a measurable change in the device conductance, off current and surface charge density values extracted from the FET transfer curves that are proportional to acetic acid concentration. Furthermore, we showed that sensor responses based on values of off current and surface charge density are selective to the type of surface interaction. Such responses will play a vital role in forming the multi-dimensional input required for the next generation of InAs NWFET sensors.

## Figures and Tables

**Table 1:** Summary of analyte properties and FET transport parameters while exposed to the analyte.

Analyte	Formula	*P*_v_^a^ (kPa)	*V*_Th_ (V)	*I*_off_ (µA)	μ_FE_ (cm^2^/V s)	*S*_S_ (V/dec)	*Q*_S_ (*e* cm^−2^)
nitrogen	N_2_	–	−1.6 to −1.8	0.12 to 0.42	540 to 770	1.6 to 2.2	1.3 to 1.6 × 10^13^
methanol	CH_3_OH	14	−1.8	0.12	440	1.9	1.6 × 10^13^
2-butanone	CH_3_COC_2_H_5_	25	−2.1	0.15	610	1.7	1.6 × 10^13^
acetic acid	CH_3_COOH	1.8	–	–	–	–	–
$P/P_{v}$ = 0.09	–	–	–	1.6	50	13.6	1.0 × 10^14^
= 0.15	–	–	–	16	110	23.6	1.8 × 10^14^
= 0.21	–	–	–	25	110	29.1	2.2 × 10^14^

^a^ Equilibrium vapour pressure at 295 K.

## Supplementary Materials

The following are available online at www.mdpi.com/1424-8220/17/7/1640/s1: Figure S1: Transient current in acetic acid at $V_{\mathrm{GS}}=-1V$, Figure S2: Semi-log plots of transfer curves, Figure S3: Continuous transient current measurement from Figure 3.
