# Supplementary material for: Sensing Responses Based on Transfer Characteristics of InAs Nanowire Field-Effect Transistors
Source: Sensors (Basel). 2017 Jul 16;17(7):1640. doi: 10.3390/s17071640 (PMC5539772; doi:10.3390/s17071640)
Supplement: Supplementary file 1 [file sensors-17-01640-s001.pdf]

Supporting Information for:

"Sensing Responses Based on Transfer Characteristics of InAs Nanowire Field-Effect Transistors"

Authors:

Alex C. Tseng [1,2], David Lynall [1,2], Igor Savelyev [1], Marina Blumin [1], Shiliang Wang [3] and Harry E. Ruda [1,2]

[1] Centre for Advanced Nanotechnology, University of Toronto, 170 College Street, Toronto, Ontario M5S 3E4, Canada

[2] Department of Materials Science and Engineering, University of Toronto, 184 College Street, Toronto, Ontario M5S 3E4, Canada

[3] Defence Research and Development Canada Suffield, Medicine Hat, Alberta T1A 8K6, Canada

Transient Current in Acetic Acid at V_{GS} = -1V

Figure S1 shows a sensing experiment performed with acetic acid and an InAs multi-NWFET. The top trace shows the relative vapour concentration set-point of the rotameters during the exposure. In this experiment, the back-gate was set to a constant DC bias of -1V, which was the threshold voltage for this device.

All the features of the exposure as discussed in Section 3.3 of the main text are present—that is, an initial drop in current followed by a gradual rise. However, in this case, the baseline continues to rise significantly past the initial values of source-drain current. We believe this to be due to a constant variation in the nanowire capacitance due to adsorption. This change in capacitance alters the electrostatic coupling of the back-gate to the NW channel, which leads to a change in operating point of the FET. In this case, it becomes difficult to compare the changes in conductance at different vapour concentrations, since the change in electron density is affected by both the change in gate coupling and the influence of adsorption.

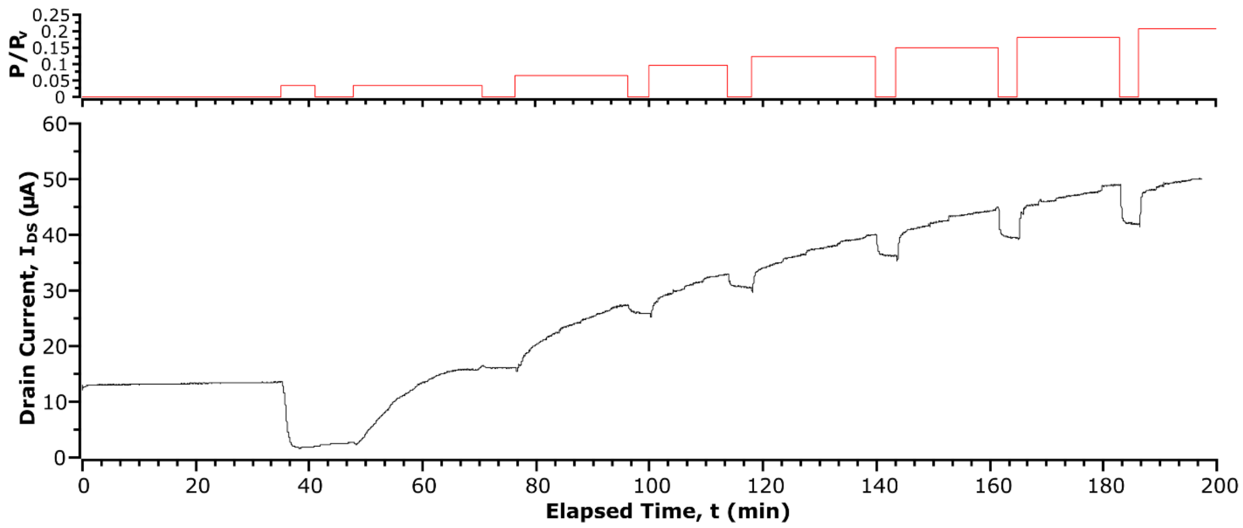

Figure S1: I_{DS} versus elapsed time of a sensing experiment with applied back-gate potential (V_{GS} = -1V). The top curve represents the relative vapour concentration as set by the flow rates through the rotameters. The bottom curve is the transient current measurement.

Semi-log Plots of Transfer Curves

Figure S2 shows the transfer curves presented in the Sections 3.1 and 3.2 of the main text plotted with a log scale for I_{DS}. From these plots, the value of subthreshold swing (S_S) can be extracted as the inverse of the slope of the log-linear region of the transfer curve. Additionally, the effect of analyte adsorption on values of I_{off} are readily apparent on this scale.

The analysis in Section 3.2 extracts values of S_S for the transfer curves in acetic acid. While it is not clear that these curves show a change to subthreshold behaviour (and a value for V_{Th}), we observe a slight inflection which is coincident with the shoulder of the curves in N_2. The shoulder indicates the change to subthreshold regime, and, in acetic acid, we take this as an indication that the transfer behaviour of the underlying NW is being masked by the effect of adsorption. Hence, the same values of V_{Th} as in N_2 are employed in the formula calculations.

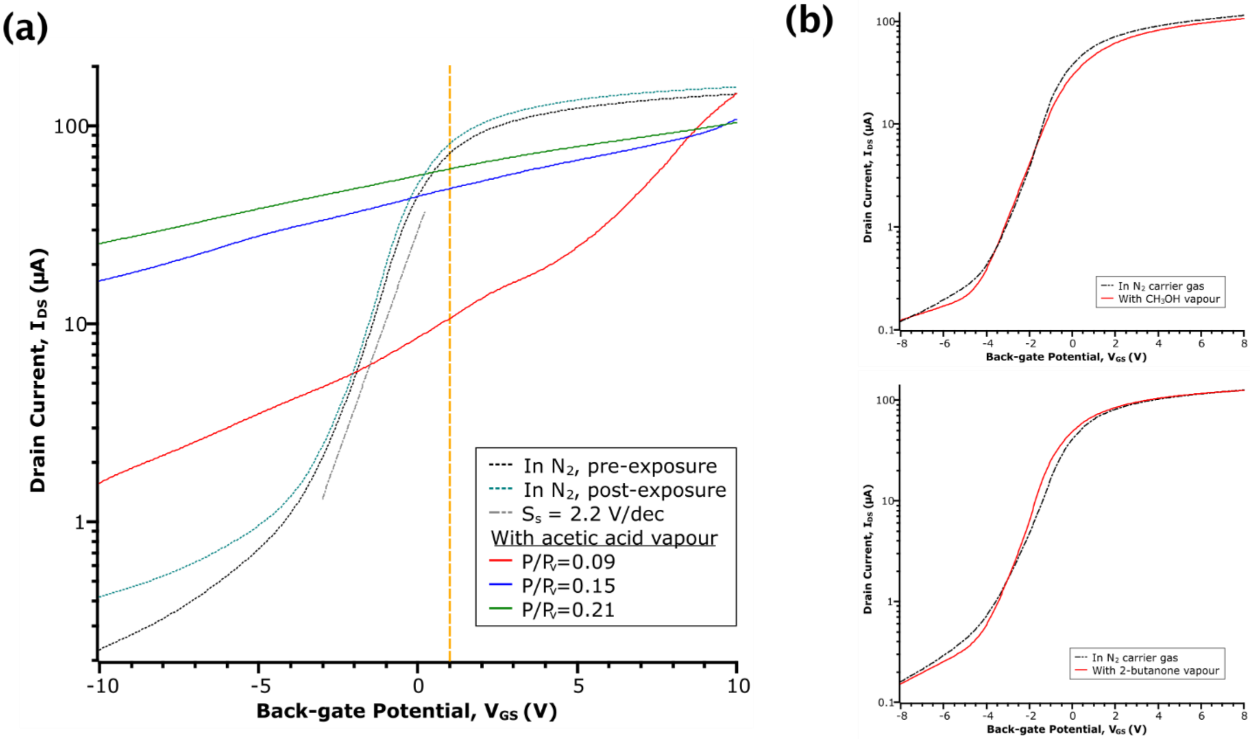

Figure S2: Data from (a) Figure 1a and (b) Figure 2 from the main text plotted on a log scale for I_{DS}. In (a), the grey dashed line has a slope of 2.2 V/dec, showing the extraction of S_S from the transfer curve in N_2. The orange dashed line is a guide to the eye to show the coincidence of the inflection point in the N_2 curve with a change in slope of the acetic acid curves.

Continuous Transient Current Measurement from Figure 3

Figure S3 shows the continuous current-time trace that was shown in cropped form in Figure 3 of the main text. As in Figure S1, the top trace shows the relative vapour concentration used to set the rotameters. In the second period (ca. 100 – 120 min), the rotameter deviated low from its set point for an extended length of time before correction. However, the current response thereafter agrees with the trend from other periods exposed to the same vapour concentration.

The three curves shown in Figure 3b are taken from the 4th to 6th period (ca. 170 – 230 min), as the timing of the exposures are the most similar. That is, in these three instances, the time spent in N_2 prior to exposure, the time exposed to acetic acid vapour, and the time required for the device to return to a baseline after removal of vapour from the chamber.

In the third period of exposure (ca. 140 – 150 min), we observe a greater drop in current once acetic acid is added to the chamber. This is likely related to the greater amount of time spent in N_2 as compared to other periods of exposure (i.e. 20 min vs. 10 min). In the main text of the paper, this is attributed to time allowed for desorption of hydrogen from the acid-base reaction, and thus the re-activation of some surface sites for the proposed reaction.

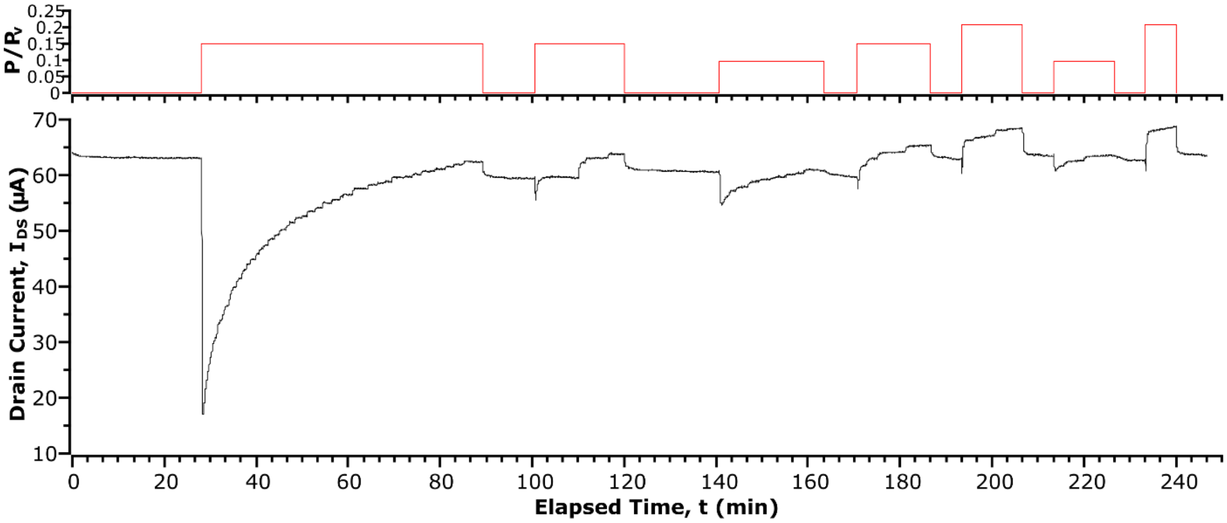

Figure S3: I_{DS} versus elapsed time of the sensing experiment shown in Figure 3 of the main text. The top curve represents the relative vapour concentration as set by the flow rates through the rotameters. The bottom curve is the transient current measurement.
